# Evidence of Immunosuppressive and Th2 Immune Polarizing Effects of Antidiabetic* Momordica charantia* Fruit Juice

**DOI:** 10.1155/2017/9478048

**Published:** 2017-07-25

**Authors:** Rufine Fachinan, Adnette Fagninou, Magloire Pandoua Nekoua, Abdou Madjid Amoussa, Marius Adjagba, Latifou Lagnika, Anatole Lalèyè, Kabirou Moutairou, Akadiri Yessoufou

**Affiliations:** ^1^Laboratory of Cell Biology and Physiology, Department of Biochemistry and Cellular Biology, Faculty of Sciences and Technology (FAST) and Institute of Applied Biomedical Sciences (ISBA), University of Abomey-Calavi, 01 BP 918 Cotonou, Benin; ^2^Laboratory of Biochemistry and Bioactive Natural Substances, Faculty of Science and Technology, University of Abomey-Calavi, Cotonou, Benin; ^3^Laboratory of Human Cytogenetics, UFR of Human Biology, Faculty of Health Sciences, University of Abomey-Calavi, Cotonou, Benin

## Abstract

The mechanism of action of the antidiabetic capacity of* Momordica charantia* is still under investigation. Here, we assessed phytochemical compositions, antioxidant activity, and effects of total and filtered fruit and leafy stem juices of* Momordica charantia* on human T cell proliferation and differentiation through quantification of Th1/Th2 cytokines. In the absence of stimulation, total fruit and leafy stem juices induced significant T cell proliferation. Under PHA stimulation, both juices potentiated plant-induced T cell proliferation. However, the filtered fruit and leafy stem juices significantly inhibited PHA-stimulated T cell proliferation, while neither juice influenced T cell proliferation. Moreover, total and filtered fruit juice increased IL-4 secretion, while total and filtered leafy stem juice enhanced IFN-*γ* production. Phytochemical screening revealed the presence of tannins, flavonoids, anthocyans, steroids, and triterpenoids in both juices. Alkaloids, quinone derivatives, cardenolides, and cyanogenic derivatives were undetectable. The saponins present in total juices were undetectable after filtration. Moreover, both juices had appreciable antioxidant capacity. Our study supports the type 1 antidiabetic effect of filtered fruit juice* of M. charantia* which may be related to its immunosuppressive and T-helper 2 cell inducing capacities. Due to their immune-stimulatory activities and their ability to increase T-helper 1 cell cytokines, total fruit and leafy stem juices may serve in the treatment of immunodeficiency and certain infections.

## 1. Background

The use of local medicinal plants is increasingly becoming a priority in developing countries, so that some of these countries have made it a privileged area of research. Indeed, populations of these countries often rely on herbal concoctions for primary health care as an alternative option to modern synthetic drugs that are more costly.* Momordica charantia* is a plant commonly used in Mediterranean traditional medicine for its antidiabetic properties and antihyperglycemic, antitumor, anti-inflammatory, and cytotoxic activities [1–4].* Momordica charantia*, also called “bitter melon” or African cucumber, is a plant of Cucurbitaceae family widely cultivated in tropical and subtropical regions. This plant is used in the countries of South Asia, South America, and the East as a vegetable or medicinal plant. The extracts of the various components of this plant (stem, leaf, fruit, and seeds) have been found to have various medicinal properties, in particular the inhibition of protein synthesis [5–7] and antitumoral and antimutagenic activities [8–10]. In addition,* Momordica charantia* fruit juice has been shown to induce regeneration of pancreatic beta cells in streptozotocin- (STZ-) induced diabetic rats [11]. Moreover,* Momordica charantia* (karela) fruit extract has exhibited hypotriglyceridemic and hypocholesterolemic antidiabetic effects in STZ-induced diabetic rats [12]. It also significantly stimulated both the storage of glycogen in the liver [13] and insulin secretion by *β*-cells isolated from the islets of Langerhans [14]. Nonetheless, the exact mechanism of action of this plant remains unclear. It has been proven that all forms of diabetes have been linked to a pathological role of the immune system and inflammation [15, 16] which implicates T lymphocytes, the principal mediators of immune responses in health and disease. Indeed, type 1 diabetes involves autoimmune destruction of pancreatic beta cells by autoreactive T lymphocytes [16] through cytokines or cell-cell contact. It is well known that naïve T-helper cell (Th0) can differentiate into several specific subsets (Th1, Th2, Th9, Th17, Th22, Treg, etc.) under the influence of cytokines [17]. The cytokines can either promote T cell differentiation into specific subsets or block differentiation towards functionally opposing subsets [17]. Th1 cells, producing proinflammatory cytokines (IL-2, IL-12, and IFN-*γ*), support cell-mediated immunity and as a consequence promote inflammation, cytotoxicity, and delayed-type hypersensitivity, whereas Th2 cells, secreting anti-inflammatory cytokines (IL-4, IL-5, and IL-13), support humoral immunity and downregulate the inflammatory actions of Th1 cells [18–20]. IL-10 appears, more and more, as a regulatory cytokine produced by several types of cells including Treg cells, CD4+ Teff cells, and Breg cells [21–25]. Thus, we hypothesized that an intervention on T cell activation and differentiation would be a valuable tool to disrupt disease progression. The beneficial effect of* Momordica charantia* in diabetes could be through its action on the immune system. Therefore, the aim of this study was to investigate the effect of the juices from different parts of this plant (fruit juice and leafy stem) on the activation and differentiation of human T lymphocytes.

## 2. Materials and Methods

### 2.1. Plant Materials' Description and Collection

Plant materials of* Momordica charantia* were collected from the south-eastern part of Benin (Adjarra city, Department of Ouémé) during the short dry season which extends from mid-July to mid-August when the mean temperature is 28.2°C (ASECNA, Air Navigation and Security Agency, Station of Dangbo, Ouémé). This period is preceded by the long rainy season (mid-March to mid-July). The soil is hydromorphic lateritic on clay sediments (reference: Carte pédologique de reconnaissance à 1/200,000 Feuille de Porto-Novo 1975, Benin) and the plant adapts to this kind of soil. Plants were identified by the Principal Botanist of the National Herbarium of Benin of the University of Abomey-Calavi, where the voucher specimens were deposited under the following number:* Momordica charantia* L., Cucurbitaceae: AP2033/HNB.

### 2.2. Plant Juices' Preparation

Leafy stems and fresh fruits of* Momordica charantia* were collected and used to prepare juices. Briefly, 100 g of leafy stems was manually ground and pressed with 100 ml of distilled water to obtain leafy stem juice. Fruit juice was obtained from fresh fruits (100 g of fruit in 100 ml of water) using slightly modified methods of Raza et al. [11]. The debris was removed by passing each mixture through a clean cotton column in a funnel. The obtained liquids representing total fruit juice or leafy stem juice were distributed in aliquots and frozen at –80°C for future use in the study. To obtain filtered juices, total juices were then filtered on filter paper (Prolabo filter paper for Ashless analysis, diameter 150 mm, Paris).

### 2.3. T Cell Isolation and Preparation for Culture

Peripheral blood mononuclear cells (PBMC) were isolated from whole blood of healthy donors as described elsewhere [26], using Ficoll solution and centrifugation. Briefly, PBMC were removed from blood diluted (v/v) with RPMI-1640 medium (BioWhittaker, Liege, Belgium) supplemented with 2 mM L-glutamine, 100 U/ml of penicillin, and 100 *μ*g/ml of streptomycin and 25 mM HEPES. Diluted blood was delicately deposited above the Ficoll solution. After centrifugation (1800 rpm, 30 min, room temperature), PBMC were removed, washed, and prepared for culture. Cell number and viability were assessed by the trypan blue exclusion test. Blood removal from human was conducted in accordance with the 1964 Declaration of Helsinki (as revised in Edinburgh 2000). The volunteers understood the study and gave their consent. The protocol was approved by the official Ethical Committee under the number Dec.n°075/CER/ISBA-2015.

### 2.4. Effects of Plant Juices on T Cell Blastogenesis

To Investigate the effects of plant juices on T cell proliferation, freshly isolated T cells were suspended in RPMI-1640 medium, seeded in 96-well round bottom plate (TPP, tissue culture testplate 96U, Switzerland), and cultured in the presence of 5 *μ*g/ml of phytohemagglutinin (PHA, Gibco, USA) used as mitogen that induces the proliferation of responder cells. Prior to use in cell cultures, plant juices were filtered [filter 0.20 *μ*m (Nalge Nunc International Corp., USA)] to avoid contamination. Cell culture was performed in the presence of increasing concentrations of plant juices (10 *μ*g/ml, 50 *μ*g/ml, and 100 *μ*g/ml). Cells were distributed in six replicates as follows: 160 *μ*l of cell suspension (10^5^ cells), 20 *μ*l of plant juice, and 20 *μ*L of mitogen or RPMI-1640 medium, as previously described [26, 27]. Plates were incubated for 6 days at 37°C in a humidified chamber containing 95% air and 5% CO_2_. At the end of the incubation period, cell culture supernatants were removed and frozen at –80°C for future determination of T cell-derived cytokine concentrations (see below). Then, cell counting was performed using trypan blue exclusion test on microscopy.

### 2.5. Determination of Th1/Th2 Cytokines in Cell Culture Supernatants

To evaluate the effects of plant juices on T-helper cell phenotype, the concentrations of T cell differentiation cytokines (IL-2, IL-4, IL10, and IFN-*γ*) in cell culture supernatants (cell cultures were described above) were quantified, using BioLegend's human Th1/Th2 ELISA MAX™ Deluxe set kits (BioLegend, San Diego, CA, USA), according to the manufacturer's instructions.

### 2.6. Phytochemical Analysis of the Plant Juices

We assessed the chemical compounds of plant juices (Table 1), using the methods of Ciulei [28], based on colorimetric reactions and differential precipitations. Briefly, total plant juices or filtered juices of leafy stem and fresh fruits were evaporated under vacuum at 50°C (Rotavapor) to obtain powders which were subjected to the determination of different compounds as previously described [28].

### 2.7. Radical Scavenging Activities of Plant Extracts

The antioxidant status of plant juices was assessed by determining the ability of juices to scavenge a free radicals' generator, the 2,2-diphenyl-1-picrylhydrazyl (DPPH) radical. DPPH is a stable free radical because of its spare electron delocalization over the whole molecule. It is commonly used to determine the antioxidant activity of various compounds. This method is based on the reduction of DPPH in the presence of a hydrogen-donating antioxidant, inducing a color change from purple to yellow at 517 nm on spectrophotometer (VWR UV1600PC Spectrophotometer, China). The degree of reduction in absorbance measurement indicates the radical scavenging (antioxidant) juice of the plant. The antioxidant activity was determined according to the method previously described [29]. Briefly, 1.5 ml of a freshly prepared methanolic solution of DPPH (2%) was mixed with 0.75 ml of juice solution (100–0.78 *μ*g/ml). After 30 min of incubation in the dark at room temperature, absorbencies were measured at 517 nm against a blank sample consisting of a 1.5 ml of methanol and 0.75 ml of juice solution. All tests were performed in triplicate. DPPH radical inhibition percentage was calculated according to the following formula: inhibition (%) = [(*AB*–*As*)/*AB*] × 100, where *As* is the sample (tested extract solution) absorbance and *AB* is the blank absorbance.

### 2.8. Determination of Total Flavonoid Content of the Juices

Flavonoid contents of the juices were determined according to the colorimetric assay described previously [30]. Briefly, 3 ml of methanol, 0.2 ml of 1 M potassium acetate, 0.2 ml of 10% aluminium chloride, and 5.6 ml of distilled water were added to 1 ml of juice (100 *μ*g ml^−1^). After half an hour of incubation at room temperature, the absorbance of the mixture was read at 415 nm using UV spectrophotometer. Quercetin was used as reference compound to produce the standard curve (*y* = 0,325*x*  −  0,363; *R*2 = 0,995) and the results were expressed as mg of quercetin equivalent (QE)/100 mg of juices.

### 2.9. Statistical Analysis

Data are expressed as mean ± SEM. Mean values were compared by two-way ANOVA, followed by LSD test. Differences were considered significant when *p* < 0.05.

## 3. Results

### 3.1. *Momordica charantia* Juices Modulate T Cell Proliferation

**Figure 1:**
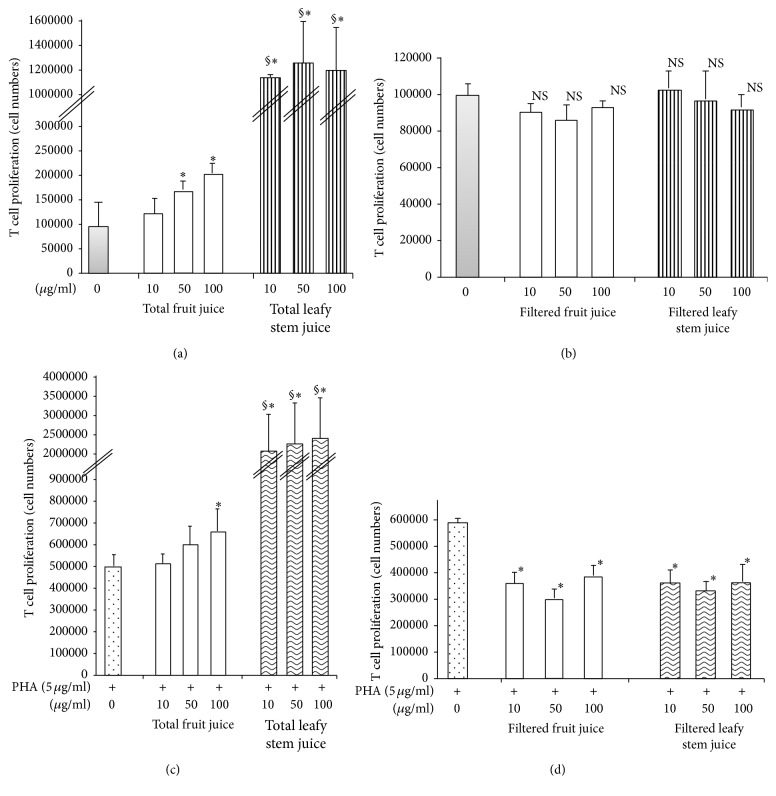
Effects of plant juices on human T cell proliferation.  Figure 1 shows the effects of plant juices on human T cell proliferation. Each value represents the mean of six determinations. Values are means ± SEM. (a) Cells cultured without mitogen and in presence of total juices; (b) cells cultured without mitogen and in presence of filtered juices; (c) PHA-stimulated cells in presence of total juice; (d) PHA-stimulated cells in presence of filtered juices. *∗* indicates significant difference between cells cultured in presence of plant juices and cells cultured in presence of medium only (*p* < 0.05). § indicates significant difference between cells cultured in presence of leafy stem juice and cells cultured in presence of fruit juice (*p* < 0.05). NS: nonsignificant difference.

To test the effect of plant juices on T cell proliferation, we stimulated human T cell with or without PHA in the presence of increasing concentration of plant juices. We observed that, without any additional stimulation, total fruit and leafy stem juices induced a significant T cell proliferation with apparent dose-dependence. The effects of fruit juice seem to be in a dose-dependent manner (Figure 1(a)). In contrast, filtered juices did not significantly influence T cell proliferation in the absence of PHA stimulation (Figure 1(b)). In combination with PHA stimulation, total juices increased T cell proliferation as compared to untreated stimulated T cell (Figure 1(c)). However, the filtered juices significantly inhibited PHA-stimulated T cell proliferation (Figure 1(d)).

### 3.2. Fruit and Leafy Stem Juices Modulate In Vitro Cytokine Production by T Lymphocytes

#### 3.2.1. Interleukin-2 (IL-2)

**Figure 2:**
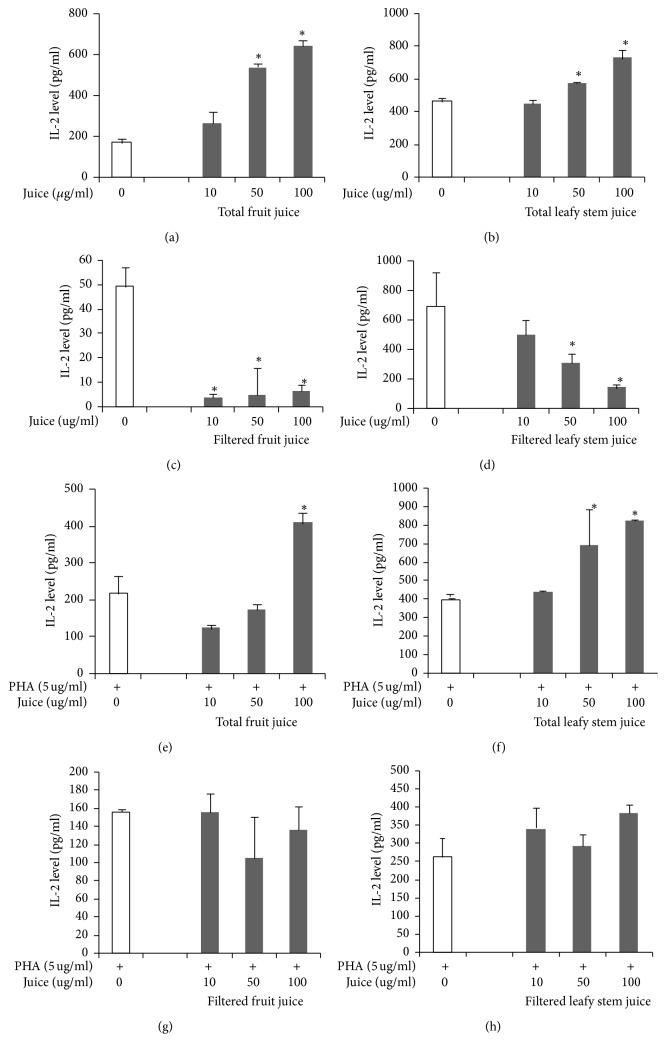
Th1 (IL-2) cytokine concentrations in cell culture supernatants in the presence of plant juices. Cytokine concentrations were determined as described in Materials and Methods. Each value represents the mean of triple determinations. IL-2 concentrations in supernatants of cells cultured without mitogen and in presence of total fruit juice (a), total leafy stem juice (b), filtered fruit juice (c), and filtered leafy stem juice (d). IL-2 concentrations in supernatants of PHA-stimulated cells cultured in presence of total fruit juice (e), total leafy stem juice (f), filtered fruit juice (g), and filtered leafy stem juice (h). Values are means ± SEM. ^*∗*^*p* < 0.05 indicates significant difference between plant juices and controls.

Total fruit juice and total leafy stem juice induced an increased secretion of IL-2 in a dose-dependent manner (Figures 2(a) and 2(b)). However, filtered fruit juice and leafy stem juice, in a dose-dependent manner, induced a significant decrease of IL-2 levels (Figures 2(c) and 2(d)). Under PHA stimulation, total fruit juice and total leafy stem juice enhanced the secretion of IL-2 (Figures 2(e) and 2(f)). In contrast, filtered fruit juice or leafy stem juice did not induce a significant change in IL-2 secretion (Figures 2(g) and 2(h)).

#### 3.2.2. Interferon-*γ* (IFN-*γ*)

**Figure 3:**
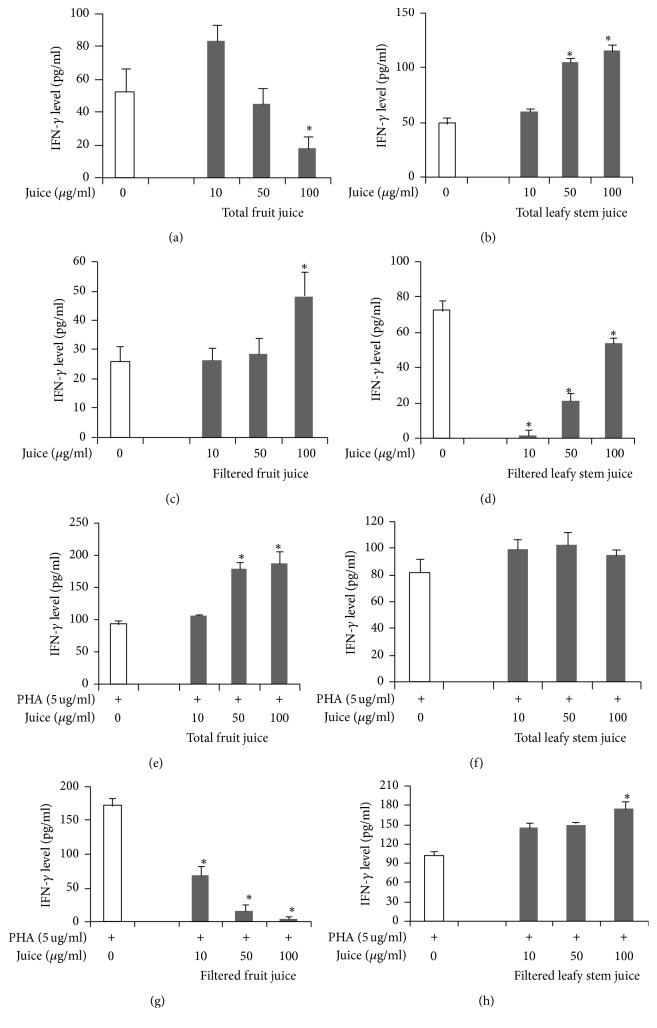
Th1 (IFN-*γ*) cytokine concentrations in cell culture supernatants in the presence of plant juices. Cytokine concentrations were determined as described in Materials and Methods. Each value represents the mean of triple determinations. IFN-*γ* concentrations in supernatants of cells cultured without mitogen and in presence of total fruit juice (a), total leafy stem juice (b), filtered fruit juice (c), and filtered leafy stem juice (d). IFN-*γ* concentrations in supernatants of PHA-stimulated cells cultured in presence of total fruit juice (e), total leafy stem juice (f), filtered fruit juice (g), and filtered leafy stem juice (h). Values are means ± SEM. ^*∗*^*p* < 0.05 indicates significant difference between plant juices and controls.

Total fruit juice, in a dose-dependent manner, decreased the in vitro secretion of IFN-*γ*, while total leafy stem juice significantly increased the same cytokine (Figures 3(a) and 3(b)). In contrast, filtered fruit juice increased and filtered leafy stem juice decreased IFN-*γ* production by T lymphocytes (Figures 3(c) and 3(d)). Under PHA stimulation, total fruit juice increased the secretion of IFN-*γ* in a dose-dependent manner, while total leafy stem juice decreased the production of the same cytokine (Figures 3(e) and 3(f)). Moreover, we observed a decrease in IFN-*γ* secretion by PHA-stimulated cells treated with filtered fruit juice and an increase in IFN-*γ* secretion by cells treated with filtered leafy stems in a dose-dependent manner (Figures 3(g) and 3(h)).

#### 3.2.3. Interleukin-4 (IL-4)

**Figure 4:**
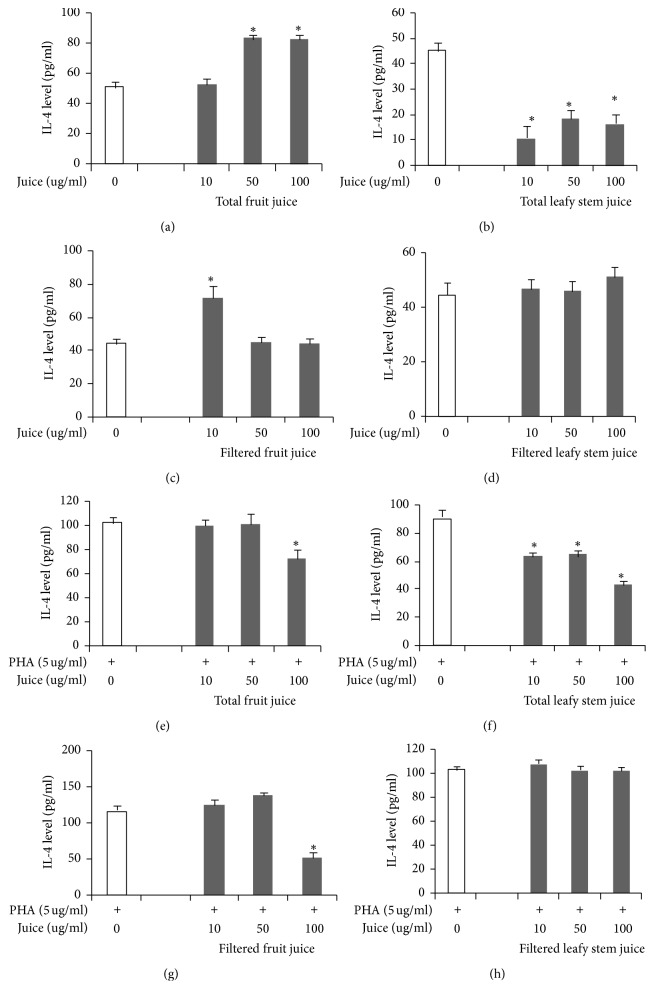
Th2 (IL-4) cytokine concentrations in cell culture supernatants in the presence of plant juices. Cytokine concentrations were determined as described in Materials and Methods. Each value represents the mean of triple determinations. IL-4 concentrations in supernatants of cells cultured without mitogen and in presence of total fruit juice (a), total leafy stem juice (b), filtered fruit juice (c), and filtered leafy stem juice (d). IL-4 concentrations in supernatants of PHA-stimulated cells cultured in presence of total fruit juice (e), total leafy stem juice (f), filtered fruit juice (g), and filtered leafy stem juice (h). Values are means ± SEM. ^*∗*^*p* < 0.05 indicates significant difference between plant juices and controls.

Total fruit juice significantly increased IL-4 secretion, while total leafy stem juice decreased the same cytokine in a dose-dependent manner (Figures 4(a) and 4(b)). Also, the maximal secretion of IL-4 observed in T cells incubated with 10 *μ*g/ml of filtered fruit juice decreased to the normal values with concentrations of 50 and 100 *μ*g/ml. At 10 and 50 *μ*g/ml, filtered leafy stem juice did not induce any change in IL-4 secretion; however, at 100 *μ*g/ml, there was a slight but significant increase in IL-4 secretion (Figures 4(c) and 4(d)).

Under PHA stimulation, IL-4 secretion, which did not change at 10 to 50 *μ*g/ml of total fruit juice, dramatically decreased in cells treated with 100 *μ*g/ml of fruit juice. Whatever the dose, total leafy stem juice totally curtailed the secretion of IL-4 in PHA-stimulated T cells (Figures 4(e) and 4(f)). On the other hand, filtered fruit juice and leafy stem juice, in a dose-dependent manner, respectively, increased and decreased IL-4 secretion by T cells; but we observed maximal effects at 10 *μ*g/ml of leafy stem and at 100 *μ*g/ml of fruit juice (Figures 4(g) and 4(h)).

#### 3.2.4. Interleukin-10 (IL-10)

**Figure 5:**
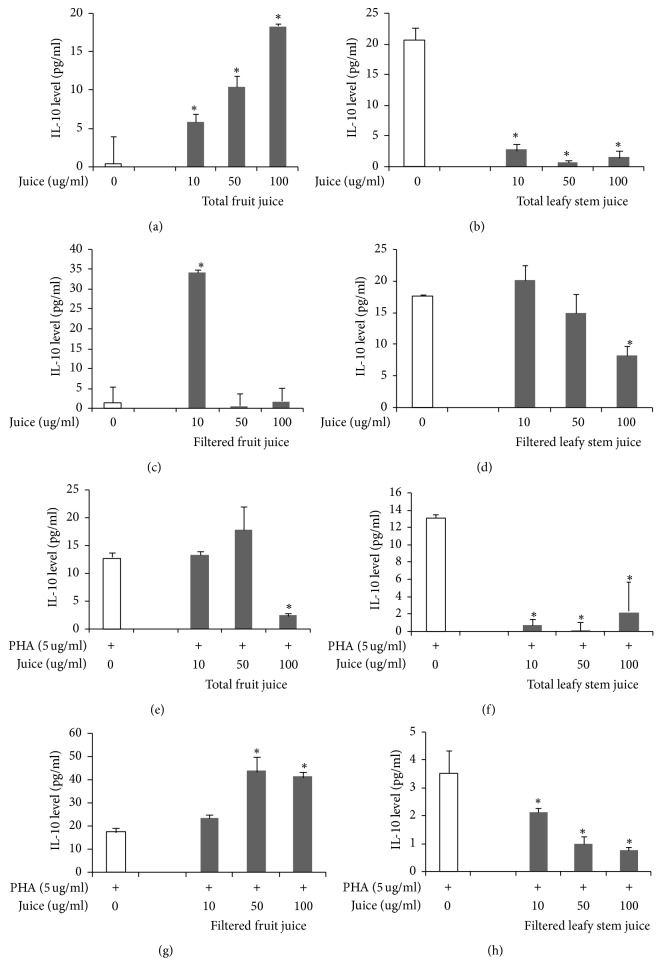
Th2 (IL-10) cytokine concentrations in cell culture supernatants in presence of plant juices. Cytokine concentrations were determined as described in Materials and Methods. Each value represents the mean of triple determinations. IL-10 concentrations in supernatants of cells cultured without mitogen and in presence of total fruit juice (a), total leafy stem juice (b), filtered fruit juice (c), and filtered leafy stem juice (d). IL-10 concentrations in supernatants of PHA-stimulated cells cultured in presence of total fruit juice (e), total leafy stem juice (f), filtered fruit juice (g), and filtered leafy stem juice (h). Values are means ± SEM. ^*∗*^*p* < 0.05 indicates significant difference between plant juices and controls.

Total fruit juice, in a dose-dependent manner, induced an increased secretion of IL-10, while total leafy stem juice decreased the same cytokine (Figures 5(a) and 5(b)). However, filtered fruit juice increased the secretion of IL-10 only at 10 *μ*g/ml, whereas filtered leafy stem juice decreased IL-10 secretion in a dose-dependent manner (Figures 5(c) and 5(d)).

Under PHA stimulation, total fruit juice (100 *μ*g/ml) induced a significant decrease of IL-10 secretion, while total leafy stem juice also diminished the secretion of IL-10 (Figures 5(e) and 5(f)). Moreover, filtered fruit juice increased IL-10 secretion by PHA-stimulated cells, while filtered leafy stem juice decreased the same cytokine (Figures 5(g) and 5(h)).

### 3.3. *Momordica charantia* Juices Are Rich in Polyphenols, Steroids, and Triterpenoids

Regarding the observed effects of the extracts on T cell proliferation, we decided to analyze phytochemical composition of plant juices. In Table 1, we observed that both plant juices (fruit and leafy stem) of* Momordica charantia* are rich in steroids, triterpenoids, mucilages, and polyphenols (tannins, flavonoids, and coumarins). The saponin compounds which were present in total juices become undetectable after filtration. Alkaloids and cyanogenic derivates were not detectable in any of the juices.

### 3.4. *Momordica charantia* Juices Exhibit Radical Scavenging Activities

**Figure 6:**
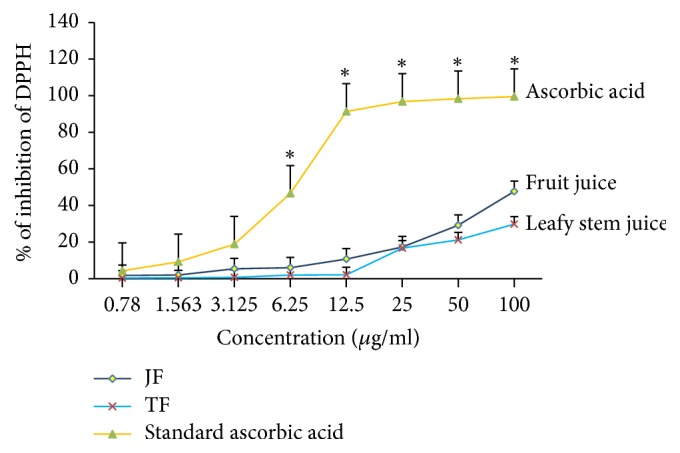
Radical scavenging activity of juice fruit* Momordica charantia*. Antioxidant capacity of plant juices was determined in a solution of juice (0.78–100 *μ*g/ml) as described in Materials and Methods. Each value represents the mean of three determinations. JF: fruit juice; TF: leafy stem juice. Values are means ± SEM. ^*∗*^*p* < 0.05 indicates significant difference between ascorbic acid values and plant juice values.

Antioxidant capacities determined as DPPH radical scavenging activities of plant juices (fruit juice and leafy stem) of* Momordica charantia* increased gradually in a dose-dependent manner (Figure 6). From 0.78 *μ*g/ml to 100 *μ*g/ml, both extracts exhibited significant antioxidant activity with high activity of fruit juice at 100 *μ*g/ml (1.78 ≤ IP% ≤ 47.65), as compared with leafy stem (0.32 ≤ IP% ≤ 29.81), even if this substantial inhibition percentage (IP%) was less than 50%, as compared to that of ascorbic acid.

### 3.5. Total Flavonoid Contents

Since the juices from fruit and leafy stem of* Momordica charantia* exhibited antioxidant activities, we determined the total flavonoids content in these juices expressed in terms of quercetin equivalent using the standard curve equation (*y* = 0.325*x* − 0.363; *R*2 = 0.995). We found that total flavonoid content of leafy stem juice of* Momordica charantia* was 22.25 ± 0.28 mg QE/100 mg, significantly higher than that of fruit juice (17.93 ± 0.17 mg QE/100 mg) (Table 3).

## 4. Discussion

The use of medicinal plants is still suffering from lack of appropriate scientific investigation that could support their inclusion in therapy. The active principles of the majority of these plants are not known and the mechanisms of action of the rare identified principles remain unclear. Although the antidiabetic properties of* Momordica charantia* are clearly established [1, 11], little is known about its mechanism of action. In this study, we aimed to investigate the effects of* Momordica charantia* juices on immune system through their effects on T cell activation. Since type 1 diabetes occurs through breakdown of self-tolerance resulting in the autoimmune destruction of insulin producing *β*-islets of pancreas by autoreactive T lymphocytes [16], we hypothesized that a modulation of T cell activation would be useful to influence disease progression. In this study, we observed that total leafy stem and fruit juices of* Momordica charantia* induced significant proliferation of human T cells in the absence of mitogen stimulation. These results are in agreement with those obtained by Ike et al. [31] who have also demonstrated in vivo that* M. charantia* pulp has shown an effective immune-stimulatory effect on Th1 cells. In the present study, the increased action of total leafy stem juice on T cell proliferation was more pronounced than that of total fruit juice. However, it is noteworthy that the filtered plant juices, without mitogen stimulation, failed to inhibit/induce cell proliferation, suggesting that filtered juices do not influence T cell proliferation under normal conditions. These results corroborate our previous findings, in which treating T cells with antidiabetic plant extracts (*Ziziphus lotus, Nauclea latifolia, Oxytenanthera abyssinica*, and* Picralima nitida*), in the absence of mitogen stimulation, did not significantly influence T cell proliferation [27, 32]. In the present study, it is interesting to observe that, in the presence of mitogen activation, total leafy stem and fruit juices potentiated their proliferative effects on T cells. However, we observed that both filtered juices, which did not influence cell proliferation under normal condition, surprisingly inhibited T cell proliferation in the presence of PHA stimulation. Then, we could suggest that the filtration has removed from the juices substances which possess double action: T cell proliferative activity and immunosuppressive-inhibiting action. The observed antiproliferative effects of filtered juices were similar to our previous findings [27, 32]. Indeed, we have found that extracts of* Ziziphus lotus, Nauclea latifolia, Oxytenanthera abyssinica*, and* Picralima nitida *have also exhibited an immunosuppressive activity on T cell proliferation [27, 32]. Similarly, Domingues et al. [33] have reported that aqueous ethanol extract of* Uncaria tomentosa*, a plant of the Rubiaceae family, significantly inhibited T lymphocyte proliferation.

As far as T cell-derived cytokines are concerned, we observed, in the present study, that the immune-stimulatory activity of total fruit and leafy stem juices was corroborated by production of IL-2 induced by these juices, regardless of mitogen stimulation (PHA). These observations appeared to be normal, since IL-2, being a growth factor, may increase T cell proliferation [34, 35].

In order to better characterize the polarity of T cells resulting from the proliferation induced by plant juices, we measured the levels of lymphocyte differentiation cytokines. General observation of these results showed that both juices of fruit and leafy stem either increased or diminished cytokine production in a dose-dependent manner. At a concentration of 100 *μ*g/ml, the effects of juices on cytokine secretion appeared to be maximal. To better appreciate the balance of cytokine production, we then evaluated the Th1/Th2 ratio at 100 *μ*g/ml of juice. We observed that Th1/Th2 ratio expressed as IFN-*γ*/IL-4 was shifted towards IL-4, a Th2 cytokine, in cells cultured in the presence of total fruit juice (Table 2), suggesting that this juice not only induced T cell proliferation but also elicited Th2-inducing capacity on T cells, in comparison to total leafy stem juice, which, on the contrary, induced a Th1-immunoproliferative effect, since leafy stem juice had shifted the IFN-*γ*/IL-4 ratio towards IFN-*γ*-producing cells. Moreover, under mitogen stimulation, we observed that both total juices increased T lymphocyte proliferation. In addition, total juices shifted the Th1/Th2 ratio towards Th1 cytokine. Our finding suggested that both juices, under mitogen stimulation, exerted a Th1-immunostimulatory effect [31]. Similar results have been observed by other investigators who demonstrated the ability of* Euclea natalensis*, an antimycobacterial and hepatoprotective plant, to increase T-helper 1 cell cytokines and to decrease the T-helper 2 cell cytokines [35].

In the case of filtered juices and without PHA stimulation, the Th1/Th2 ratio appeared to be in equilibrium in the absence of PHA stimulation. This finding was in accordance with the fact that neither juice influenced T cell proliferation. Nevertheless, mitogenic stimulation revealed the Th2 status of cells treated with filtered fruit juice and the Th1 phenotype of cells treated with filtered leafy stem juice. These findings proved that the filtered fruit juice possesses a Th2-immunosuppressive action, whereas the leafy stem juice elicited a Th1-immunosuppressive effect. The present results on the Th2-immunostimulatory effect of total fruit juice seem to be contradictory with those obtained by Ike et al. [31] who have demonstrated in vivo that* M. charantia* pulp has shown an effective immune-stimulatory effect on Th1 cells, producing IFN-*γ*. We would like to highlight that, in our study, the Th1 profile (IFN-*γ*) was also observed in cells treated with 10 *μ*g/ml of total fruit juice but the phenotype of the cells was reversed to a Th2 profile (IL4) at 100 *μ*g/ml, suggesting that the dose of juices might influence the shift of T cell phenotype.

As far as IL-10 cytokine is concerned, there was a concomitant increase or decrease of IL-10 with IL-4 levels in cells treated with both juices. These observations appear to be normal, since IL-10 is known to possess regulatory and anti-inflammatory properties [36–38].

Beneficial effects of plants are, without any doubt, related to their biologically active compounds. In attempt to understand the effects of these plant juices on T lymphocyte proliferation, we carried out the phytochemical screening of all juices. We observed that both leafy stem and fruit juices of* Momordica charantia* were rich in polyphenols (tannins, flavonoids, and coumarins), steroids, triterpenoids, and mucilages. The saponins present in crude extracts became undetectable after filtration. Alkaloids and cyanogenic derivatives were undetectable in all juices. These results are in contradiction with those observed by Johnson et al. [39] who have revealed the presence of alkaloids and free anthracene but not triterpenoids, coumarins, and saponins in* M. charantia* also collected from Benin. According to other studies,* M. charantia* should contain flavonoids, tannins, saponins, and even alkaloids [40]. It is a plant with a wide variety of active compounds [40]. This discrepancy could be related to several parameters: parts of plant used, nature of solvent, mode of preparation, geographical origin, and genetic divergences of the strains used [41]. On one hand, we could suggest that the T cell proliferative effects induced by total juices, in this study, might be related to the presence of saponins, since these compounds, present in good proportion in total juice, became undetectable after filtration. On the other hand, we could also suggest that the T cell proliferative effects of total juices might be related to the absence of alkaloids in these juices, as we have previously demonstrated that the immunosuppressive activity might be attributed to the alkaloids contained in three medicinal plants [32]. Other investigators have also proven the immunosuppressive and anti-inflammatory activity of alkaloids derivates contained in plants [42, 43]. The latter hypothesis could not be retained, since we also observed that filtered juices, which did not show alkaloids, inhibited T cell proliferation upon activation. Be that as it may, these observations proved that the immunosuppressive effects of filtered extracts cannot be attributed to the presence/absence of alkaloids. One of few reports available on the proliferative effects of saponins comes from the study of Zhang et al. [44] who have reported hormetic effect of panaxatriol saponins which stimulated PC12 cell growth by about 30% at low doses, while panaxatriol saponins at high doses inhibited cell growth. In the present study, all juices have shown the presence of polyphenolic compounds such as flavonoids, tannins, and coumarins. To our knowledge, the chemical composition of the juices did not explain their antiproliferative activity. Complementary investigations are necessary to explain these observations.

We have recently demonstrated that antidiabetic plants are often a good source of antioxidant agents [27, 32]. In this study, we observed that both extracts of* M. charantia* elicited appreciable antioxidant capacity determined as DPPH free radicals' scavenging. In this study, the polyphenolic substances (tannins and flavonoids) highlighted during phytochemical tests may explain the antioxidant capacity of both extracts (fruit juice and leafy stem). Moreover, we found that fruit juice and leafy stem extracts of* Momordica charantia* exhibited good quantity of total flavonoid, confirming the observed antioxidant activity of both extracts revealed by this study. The tannins act as scavengers of free radicals which are produced during lipid oxidation [45]. It has been observed that many flavonoids and tannins exhibit antioxidant activities, detoxification, and numerous health promoter effects such as anti-inflammatory and anti-diabetic effects [45].

## 5. Conclusion

To sum up, our results confirm the antioxidant activity of* Momordica charantia*. The novel finding of our study is that total fruit juice elicited a Th2-immunoproliferative capacity, and saponins-depleted fruit juice exhibited an immunosuppressive activity and Th2 inducing capacity on T cells, which may contribute to inhibiting deleterious effect of autoreactive T lymphocytes on *β*-cells. These observations agreed with the beneficial effect of this plant in autoimmune type 1 diabetes reported by Ahmed et al. [46] who have shown that oral feeding of* M. charantia* fruit juice may have a role in the renewal of pancreatic *β*-cells in STZ-diabetic rats or alternately may permit the recovery of partially destroyed *β*-cell. Besides, the Th2 profile of cells induced by fruit juice suggests and confirms the anthelmintic properties reported for this plant [47]. On the other hand, leafy stem juice showed Th1-immune-stimulatory and proinflammatory potency and could be useful in the treatment of certain intracellular parasite and microbial infections [47].

## Figures and Tables

**Table 1:** Phytochemical compositions of plant juices.

Chemical compounds class	Test	*Momordica charantia* (part used)			
		Leafy stem		*Fruit*	
		Total juice	Filtered juice	Total juice	Filtered juice
Alkaloids	General test: Dragendorff reagent	−	−	−	−
	Extraction: Mayer reagent	−	−	−	−

Tannins	Few drips of FeCl3, 1%	+++	+++	+++	++

Flavonoids	Adding four drips of HCl 5% to 1 ml of juice	+	+	+	+

Saponins	Foam index (FI) of diluted aqueous decoction (positive if FI ≥ 100, meaning foam height ≥ 1 cm)	++ (FI > 1 cm)	− (FI < 1 cm)	++ (FI > 1 cm)	− (FI < 1 cm)

Triterpenoids	Liebermann-Burchard reaction (acetic anhydride-sulfuric acid 50 : 1)	++	+	+	+

Mucilages	Viscosity study (in absolute ethanol)	+	+	+	+

Coumarins	Addition of 0.5 ml of NH_4_OH 10%	+	+	++	++

Anthraquinones	Addition of 1 ml NH_4_OH 25% + 1 ml NaOH	+	−	−	−

Steroids	Acetic anhydride-chloroform + concentrated sulfuric acid	+	+	+	+

Cyanogenic derivates	Grignard reaction soaked paper with picric acid 5%	−	−	−	−

Chemical compounds of total and filtered juices. The phytochemical analysis was performed as described in Methods. +++, too high, ++, high, +, low, indicate the presence of the compounds in the juice; − indicates the absence of compound in the juice.

**Table 2:** Ratios of Th1 and Th2 cytokine concentrations in cell culture supernatants.

	Leafy stem		Fruit	
	Total juice (100 *μ*g/ml)	Filtered juice (100 *μ*g/ml)	Total juice (100 *μ*g/ml)	Filtered juice (100 *μ*g/ml)
	IFN-*γ*/IL4		IFN-*γ*/IL-4	
Ratios of cytokines from cells without mitogen	11.12	1.05	0.55	16.19
Ratios of cytokines from cell with mitogen (PHA) stimulation	3.42^*∗*^	27.07^*∗*^	6.89^*∗*^	0.12^*∗*^

The values are ratios of cytokine concentrations in supernatants of unstimulated and PHA-stimulated cells cultured in presence of 100 *μ*g/ml of plant juices. Cytokine concentrations are determined as described in Materials and Methods. ^*∗*^*p* < 0.05 indicates significant difference between both ratios with mitogen and without mitogen stimulation.

**Table 3:** Total flavonoids content in *Momordica charantia* juices.

	Total flavonoids (mgQE/100 mg)
Leafy stem juice	22.25 ± 0.28
Fruit juice	17.93 ± 0.17^*∗*^

Values are mean ± SEM (*n* = 3). Flavonoid contents of the juices were determined as described in Materials and Methods. ^*∗*^*p* < 0.05 indicates significant difference between both juices.
